# Vaccination with outer membrane vesicles from *Neisseria Meningitidis* and SBa15, SBa16 mesoporous silica associated with SARS-CoV-2 induces protective humoral and cellular response against COVID-19 in mice

**DOI:** 10.1016/j.bjid.2024.104479

**Published:** 2024-11-14

**Authors:** Bruno Gaia Bernardes, Andrew Douglas Moura, João Paulo de Oliveira Guarnieri, Carlos Fernando Macedo da Silva, Hernan Hermes M. da Costa, Ingrid Gracielle Martins da Silva, Karine Brenda Barros Cordeiro, Sônia Nair Báo, Carlos Roberto Prudêncio, Marcelo Lancellotti

**Affiliations:** a Universidade de Campinas (UNICAMP), Faculdade de Ciências Farmacêuticas (FCF), Laboratório de Biotecnologia (LABIOTEC), Campinas, SP, Brazil; b Instituto Adolfo Lutz, Centro de Imunologia, São Paulo, SP, Brazil; c Universidade de Brasília, Campus Universitário Darcy Ribeiro, Instituto de Ciências Biológicas, Departamento de Biologia Celular, Laboratório de Microscopia e Microanálise (LMM), Brasília, DF, Brazil

**Keywords:** Adjuvants, Mesoporous silica, Outer membrane vesicles, SARS-CoV-2, Vaccine, Vesicles fusion

## Abstract

The global impact of the SARS-CoV-2 (severe acute respiratory syndrome coronavirus 2) pandemic in 2019–2020 has led to significant changes in worldwide vaccination and immune prophylactic approaches. In this study, our research delves into a new immunization strategy that does not involve the use of additional adjuvants or preservatives, focusing on the effects of virus fusion with a bacterial nanostructure. The experimental procedures outlined in this paper involved the cultivation of SARS-CoV-2, the production, extraction, and nanocharacterization of outer membrane vesicles (OMV) from Neisseria meningitidis, immunization of mice with two doses of OMV combined with SARS-CoV-2, and the use of mesoporous silica SBa15 and SBa16 adsorbed to the same virus. The immune response was assessed through an indirect elisa method, analysis of cytokine expression profiles, and seroneutralization of the SARS-CoV-2 strain. The characterizations of associated OMV - SARS-CoV-2 and adsorption SBa15 and SBa16 were performed using Nanosight Tracking Analysis (NTA), which showed a high density of particles in the formulation. mice were then immunized, resulting in an immune response that produced high levels of neutralizing antibodies in IgG and IgG1 mouse immunoglobulins. In addition, expressions of IL-2, IL-4, and IL-23 in spleen cells were reinforced after the vaccination process. The comparative study of these three vaccine formulations has shown that the development of new vaccines for SARS-CoV-2 should take into consideration the production of neutralizing antibodies and the maintenance of immunological memory.

## Introduction

The global impact of the SARS-CoV-2 (Severe Acute Respiratory Syndrome Coronavirus 2) pandemic in 2019‒2020 has triggered a paradigm shift in worldwide approaches to vaccination and immune prophylactic measures. Robust humoral and cellular responses against SARS-CoV-2, observed in patients recovering from infection, have spurred collective efforts by global health organizations, notably the World Health Organization (WHO).^1^, ^2^, ^3^

The structure of SARS-CoV-2 comprises a viral envelope housing the Spike protein on its surface, along with associated proteins such as N and M within the viral capsid. These proteins play pivotal roles in immune response and activation of the immune system.^2^^,^^4^^,^^5^ Despite widespread utilization of physical and chemical prophylactic measures, including 70% alcohol and environmental disinfection with detergents and known disinfectants, these strategies proved insufficient in preventing the onset of a second wave of the pandemic chaos.

In collaboration with pharmaceutical enterprises, vaccination protocols have been established and implemented, employing diverse strategies such as RNA vaccines, inactivated whole-virus vaccines, and recombinant vaccines in adenovirus carrying foreign genetic material from SARS-CoV-2.^6^, ^7^, ^8^ This study focuses on fusion vaccines in combination with an outer membrane vesicle or proteasomes from Neisseria meningitidis,^9^ aiming to enhance the immune response and promote satisfactory immune memory in the host. Furthermore, our research explores a novel immunization strategy without the use of additional adjuvants or preservatives, examining the actual impact of virus fusion with a bacterial nanostructure. Also, we performed the evaluation of mesoporous silica associated with inactivated SARS-CoV-2 and viewed its immune response in mice models.

## Material and methods

### Cell, bacteria and SARS-CoV-2 strains

*N. meningitidis* (C2135); E6 Vero cells used in this study were obtained from (INCQS – FIOCRUZ – National Institute for Quality Control - Oswaldo Cruz Foundation, Rio de Janeiro, RJ and Cell Bank). SARS-CoV-2 B8 isolate was provided by the Adolfo Lutz Institute to determine the neutralizing titer and propagate in E6 cells. *N. meningitidis* was grown at 37 °C under 5% of CO_2_ in agar GCB (Difco). E6 cells line were cultivated in RPMI1640 medium supplemented with 10% of fetal bovine serum and 1% of antibiotics (levofloxacin 1 µg/mL, tetracycline 1 µg/mL, erythromycin 3 µg/mL in hydroalcoholic 50% solution). SARS-CoV-2 B8 was replicated in E6 Vero Cells with 70% of confluence until 75% of CPE (cytopathic effect).

### OMVs extraction and SARS-CoV-2 omv associated vaccine

OMV extraction was performed using a method of extraction by ultrafiltration (using a retention filter of 0.025 nm of diameter) following the descriptions of Martins et al., 2018.^9^ The samples were stored at −80 °C. The vaccine preparation was carried out using SARS-CoV-2 that are being released from E6 cells. For this, different concentrations (1 × 10^8^ OMV detrermined by NTA analysis) of OMV from *N. meningitidis* were added to for confluent E6 infected cells (the time of infection was 24h with a MOI = 1:100 – 1 virus particle for each 100 cells), to obtain the best vaccine preparation. The supernatants containing the, SBa15 and SBa16 adsorbed with SARS-CoV-2 and the OMV vesicles associated with SARS-CoV-2 were collected, inactivated by addition of 1% formaldehyde (v/v) and then lyophilized. The vaccine preparations were analyzed by Nanosight Tracking Analysis (NTA) and followed for mice immune responses.

### Mice experiments and ethics statement

The mice were acquired from “Centro Multidisciplinar para Investigação Biológica na Área de Ciência em Animais de Laboratório – CEMIB/UNICAMP” (http://www.cemib.unicamp.br). The protocol for animal practice was approved in accordance with relevant guidelines and regulations by the Commission on Ethics in Animal Use ‒ CEUA protocol number: 6203–1/2023 (http://www.ib.unicamp.br/comissoes/ceua). A total of 1 × 10^7^ nanoparticles were used to immunize each mouse in a subcutaneous injection volume of 0.1 mL. Double doses of subcutaneous vaccination (intervals of three weeks each one) were performed in groups of five animals. In this study, we analyzed three different vaccine formulations: 1) Control nonimmune, 2) SBa15 adsorbed with SARS-CoV-2; 3) SBa16 absorbed with SARS-CoV-2; 4) OMV in association with SARS-CoV-2 virus inactivated. The animals were maintained for more than three weeks and after anesthetizing the blood and spleen recovery for ELISA and RT-qPCR for cytokines detection.

### Measurement of anti-SARS-CoV-2 response on vaccinated mice: specific antibodies

To determine if the vaccine formulations were able to induce a protective response against SARS-CoV-2, vaccinated mice were euthanized at 14 days after vaccination by anesthesia. Total bloods of all animals were collected by heart puncture, while spleen was harvested, and used for splenocyte isolation. The presence of SARS-CoV-2 specific antibodies in the serum was evaluated by Enzyme Linked Immunosorbent Assay (ELISA), using SARS-CoV-2 coating plates containing 500 plate forming units (pfu) for each well. As a secondary antibody we used an anti-IgG mouse Whole Molecule (H+L), anti-IgG1, anti-IgM, anti-IgA all of them conjugated with peroxidase. The specific antibody response was performed as described by Martins et al. 2018.

### Presence of neutralizing antibody against SARS-CoV-2 and cytokines expression

The serum neutralizing capacity was performed following the specifications described by Da Costa et al. 2023. The cytokines expressions chemokine production induced by the vaccine formulations, splenocytes were obtained from spleen-explants and grown in RPMI1640 supplemented with calf fetal sera at 10%. The cells were incubated at 37 °C in humidity and 5% carbonic dioxide-controlled atmosphere by 4 hours and the total RNA of these cells was obtained by PureLink™ RNA Mini Kit (Ambion® Carlsbad, CA, USA). The expression of IL2, IL4, IL10, IL23, INFγ, TNFα and TGFβ were determined by relative quantification using SYBR®-one-step Reverse Transcriptase PCR system (Promega, Madison, WI, USA) after normalization with GAPDH. The reaction conditions flowed what was described above.

### Scanning electron microscopy (SEM)

The size and surface morphology of the vaccine preparations were characterized by Scanning Electron Microscopy (SEM). Samples (OMV and the association/fusion of OMV and SARS-CoV-2) were diluted with 5% (v/v) ultrapure water, and 20 μL were deposited on copper supports, dried at room temperature and coated with gold using the Blazers SCD 050® metallizer (Blazers Union AG, Liechtenstein). The samples were then analyzed using a Scanning Electron Microscope (SEM) (JEOL JSM 7001-F, Japan).

### Statistical analysis

The experiments were performed in triplicate and the results are presented as mean and standard deviation. For statistical analysis, the results were compared using 01-way analysis of variance (ANOVA), with Tukey's post-test; p-value of 0.05 indicates statistically significant differences.

## Results

**Fig. 1:**
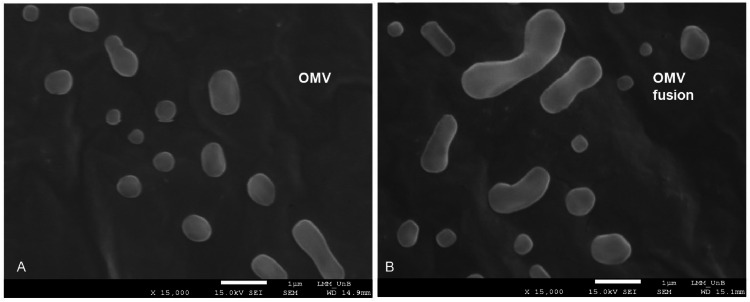
Scanning Electron Microscopy of associated nanoparticles of Outer Membrane Vesicles (OMV) from *N. meningitidis* and virus surface SARS-CoV-2. In A could see the non-associated OMV and in B associated OMV with size gain in its structure.

The characterizations of associated OMV- SARS-CoV-2 and absorption SBa15 and 16 were performed by NTA as described in Table 1. OMV and OMV associated/fused with SARS-CoV-2 were observed on scanning electron microscopy (Fig. 1). Also, the SEM of SBa15 and SBa16 were described by Amstalden et al., 2019.^10^ The results summarized in Table 1 showed that SARS-CoV-2-OMV vesicles were larger than control OMV vesicles. Additionally, a high number of nanoparticles (approximately 1.10^9^ particles/mL) was observed when analyzing the population of graphic particles, indicating the presence of two or three different populations which could affect the fusion process induced for vaccine production (data not shown). The size of OMV associated with the SARS-CoV-2 virus was also identified in Fig. 1, allowing for a comparison of the scale and size of OMV and OMV-associated particles.

**Table 1:** Characterization by NTA of the nanoparticles.

Parameter	OMV	SBa15 COVID-19 absorbed	SBa16 COVID-19 absorbed	OMV/COVID-19 association
Size (nm)	135.4 ± 11.2	199.2 ± 3.8	187.0 ± 5.6	179.9 ± 6.1
D10 (nm)	80.1 ± 2.2	114.2 ± 1.1	102.0 ± 1.2	100.1 ± 2.6
D50 (nm)	114.3 ± 6.6	178.8 ± 3.5	162.1 ± 8.9	153.4 ± 3.1
D90 (nm)	237.3 ± 28	314.7 ± 8.0	308.0 ± 3.9	321.8 ± 18.1
Particles.mL^−1^	1.79 ± 0.21 × 10^8^	2.28 ± 0.04 × 10^10^	3.58 ± 0.04 × 10^10^	1.86 ± 0.02 × 10^9^

**Fig. 2:**
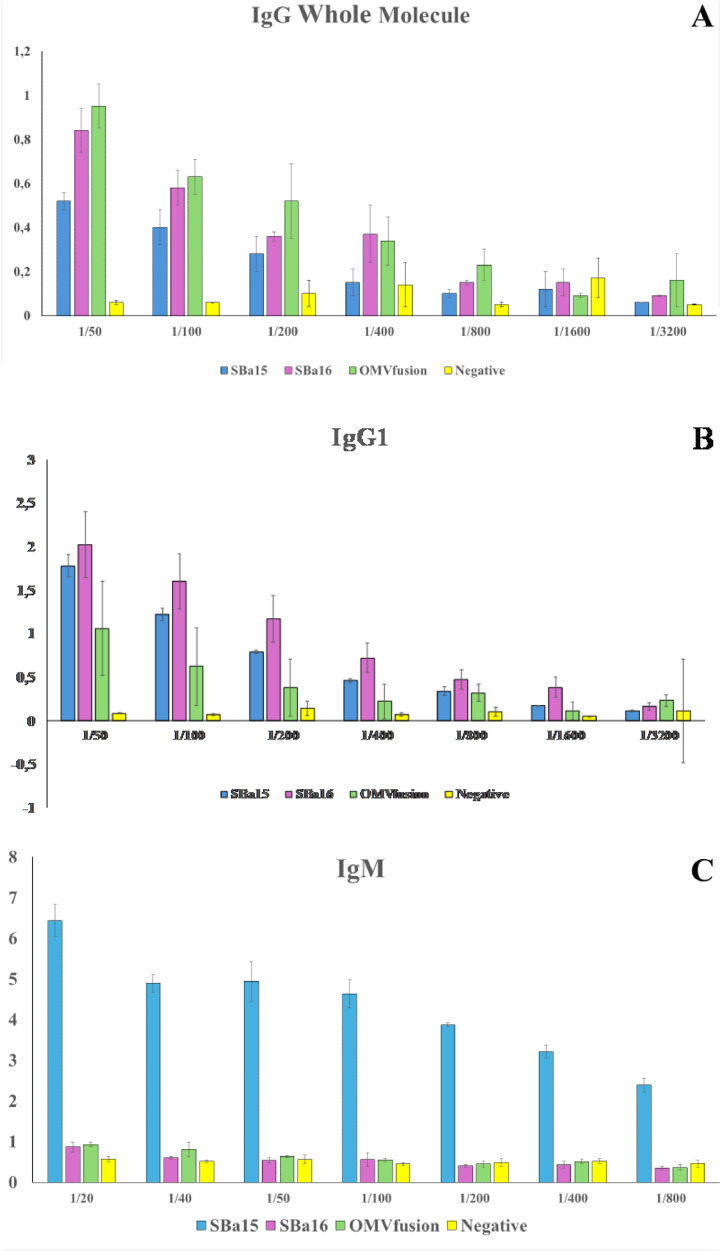
ELISA methods for antibodies production verification. In all graphics the Y axes show the absorbance values at 490 nm of antigen-antibodies reaction; and in X the values of the titer's mice serum dilution for each immunization compared with the naïve mice group. In the A ‒ IgG whole molecule detection. In B the IgG1 isoform molecule, and in C the IgM class molecules detection in each experimental group.

In the immunization process, serum antibodies were detected using ELISA methods, as described in Fig. 2A–C. We analyzed the potential of SARS-CoV-2-OMV vesicles, as well as SBa15 and 16 absorbed with the same virus, to induce the production of specific IgG, IgG1, IgA, and IgM antibodies against SARS-CoV-2. With the exception of IgA production (data not shown), all the other immunoglobulins were able to induce higher titers of IgG, IgG1, and IgM against SARS-CoV-2 on day 14 after immunization.

**Fig. 3:**
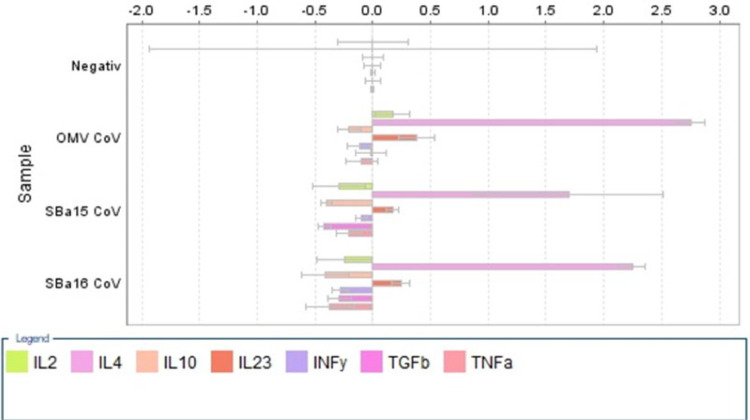
Relative expression of inflammatory cytokines in relation with GAPDH constitutive gene expression. The IL-2 (green bars) IL-4 (rose bars) and IL-23 (red bars) were over-expressed while the other cytokines were under-expressed after immunizations with vaccine preparations.

Following the analysis of humoral immunity, we examined the expression of inflammatory cytokines in the spleens of immunized mice, which are responsible for memory and quality vaccine parameters, as shown in Fig. 3. Cytokines such as IL2, IL4, IL10, IL23, INFγ, TGFβ, and TNF were assessed, with a notable increase in the expression of IL4 and IL23 in all the immunized groups. Additionally, IL2 was increased in the mice immunized with OMV associated with SARS-CoV-2, while the expression of all other cytokines decreased compared to the endogenous gene of mice spleen (GAPDH endogenous gene).

**Fig. 4:**
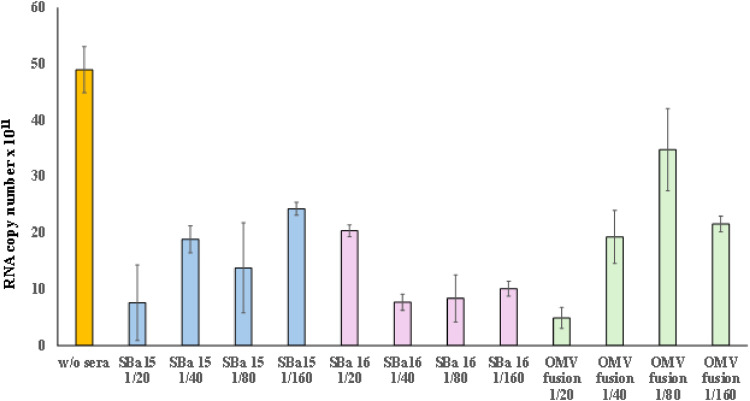
Seroneutralization of SARS-CoV-2 virus particles against vaccinated mice with SBa15 and SBa16 mesoporous silica absorbed with virus and OMV associated with SARS-CoV-2. The orange bar represents the viral copy number of the whole virus amount, and the others represent the neutralization of the half copy number of each sera titer.

Furthermore, the neutralizing effect of antibodies induced by vaccination was assessed under in vitro conditions. The Fig. 4 displayed the neutralizing effect of each serum obtained from immunized mice, showing a high capability to neutralize SARS-CoV-2. All vaccine preparations demonstrated a neutralizing effect at a dilution of 1/160, indicating the strong ability of these protocols to stimulate antibody production.

## Discussion

The use of vaccines during the COVID-19 Pandemic was one of the key factors in reducing cases and enabling a return to sustainable life, as well as the revival of economic and socio-cultural activities worldwide. Vaccination strategies played a critical role in decreasing cases and most importantly, reducing mortality resulting from various clinical conditions associated with infection by a new pathogen.^2^^,^^3^^,^^6^^,^^7^^,^^11^ In this article, we analyze the effects of new vaccine formulations that consider the use of inorganic nanostructures for adsorbing the SARS-CoV-2 virus in vitro. Specifically, we examine two different shapes of mesoporous silica with various biodistribution patterns, as well as a biological membrane nanoparticle known as OMV. Considering the differences between SBa15 and SBa16 adsorbed vaccines we aimed to verify the effects of the two forms of mesoporous silica ‒ SBa15 (tubular and hollow structure) which can promote controlled releases of substances or, in this case, viral antigens) and SBa16 (spherical/cubic shape) which can provide adjuvant of these epitopes through the release of those associated with its surface. Therefore, it is justified to compare them and to visualize different results obtained, mainly in the different production of immunoglobulin isoforms (IgM being produced by antigens associated with SBa15 and IgG1 being produced by antigens associated with SBa16.

Concerning the membrane nanoparticle associated with SARS-CoV-2 has been extensively studied by the group and has shown vaccine stimulation effects on the immune system in previous research.^12^^,^^13^ Such projections form in the membrane regions in the absence of peptidoglycans. Soluble proteins associate with Outer Membrane Vesicles (OMVs) in the periplasm and remain associated with OMVs in the external environment, acting as adhesive material. The formation of OMVs can be enhanced by antibiotics or self-lysine (which act as glucosidases, amylases, and peptidases naturally produced by bacteria during cell division).^14^, ^15^, ^16^, ^17^, ^18^, ^19^, ^20^ In this study, we compared the vaccine effects of different nanostructures and their effectiveness in immunizing mice against SARS-CoV-2. We confirmed that all preparations were able to produce neutralizing antibodies, as shown by indirect ELISA tests and serum neutralization of SARS-CoV-2 particles (as depicted in Fig. 2, Fig. 4). Additionally, the presence of a large number of particles in the preparations suggests that using both virus-adsorbed particles and those associated with OMV could be beneficial in formulating new vaccine strategies for large-scale production. Also, the values verified to the Immunoglobulin G Isoform 1 (IgG1) indicative in mice as an important marker of immunological memory acquisition, show significant values at 1/800 titers as shown in the Fig. 2B. Furthermore, the presence of an increase in the IgG1 isoform in ELISA tests for preparations containing OMV associated with SARS-CoV-2 demonstrates the memory maintenance capacity of this new vaccine.

When characterizing the nanostructures (as detailed in Table 1 and Fig. 1), we utilized the NanoSight Tracking Analysis method. This method not only provided information about the size and distribution of nanostructures but also revealed that, unlike most data in the nanotechnology literature, these nanostructures are not presented in a uniform or homogeneous manner.^9^^,^^17^^,^^21^, ^22^, ^23^ Such nano distribution is not relevant for verifying the immunological response when comparing different types of formulations. This is because antigens, whether nanostructured or not, will be metabolized by the host's immune system. Their presentation in the cells will be done through a small component of the formulation. As a result, the humoral immune response to these particles, based on their inorganic and biological natures, can be assessed for their ability to activate the immune system in the production of inflammatory cytokines.

In Fig. 3, an increase in the expression of mRNA responsible for activating interleukins 4 and 23 is observed in all vaccinated groups, whether with mesoporous silica or OMV associated with SARS-CoV-2. This increase in these two cytokines is well-documented by several authors and plays a role in both the infection and the inflammatory cytokine storm caused by SARS-CoV-2, as well as in the vaccine processes.^13^^,^^24^, ^25^, ^26^, ^27^, ^28^ Additionally, besides these two interleukins, an increase is only observed in the group vaccinated in association with OMV and SARS-CoV-2 of interleukin 2, which is commonly associated with the Th1 immune response.^4^^,^^25^^,^^29^ Like other cytokines, IL-2 also plays a role in both the inflammatory process and the vaccine response to this pathogen. Therefore, both the inflammatory processes of natural infection caused by this important and emerging pathogen and the vaccine response presented by the three different formulations in this work demonstrate similarities in the profile of antibody production, viral neutralization, and activation of the host's immune system.

**Fig. 5:**
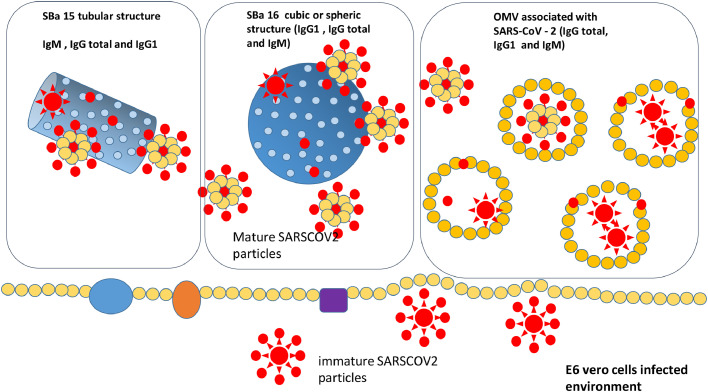
Schematic representations of the three formulations and the immunoglobulins level detected in immunized mice.

In summary, as shown in Fig. 5 the compilation of the results of this study, although it involves the development of a vaccine prototype, we believe that the combined use of different doses of the vaccine would be ideal. For example, the combination of SBa15 (which maintains IgM stimulation levels until the end of vaccination) combined with the use of the associated OMV vaccine with SARS-CoV-2 would be a first approach to be tested in future studies. Nor do we invalidate the combined studies of the three vaccine forms, whether at different doses or in combined doses.

## Conclusion

In conclusion, the comparative study of these three inorganic formulations of bacterial origin, whether in terms of their vaccine adjuvant effect or in the development of new vaccines for SARS-CoV-2, should consider both the production of neutralizing antibodies and the maintenance of immunological memory, as well as the study of the activation of the immune system and its potential adverse vaccine effects, like any other medicine in the world. The various vaccines existing in the world and distributed by different pharmaceutical companies have not taken the existence of these nanostructures into account when designing new vaccine formulations. The cost of these technologies is not a major part of the construction of this work since it is an initial project and the construction of a vaccine prototype based on organic and inorganic nanoparticles. Therefore, the discussion on costs and other particularities involving the commercial side of these vaccines will be carried out in new publications by the group in the future. However, we reaffirm the importance of research in this direction involving the association of nanostructures with SARS-CoV-2 epitopes with vaccine capacity, either as a central part of the vaccine component or as powerful adjuvants.

## Conflicts of interest

The authors declare no conflicts of interest.
